# Alpers disease mutations in human DNA polymerase gamma cause catalytic defects in mitochondrial DNA replication by distinct mechanisms

**DOI:** 10.3389/fgene.2015.00135

**Published:** 2015-04-09

**Authors:** Yufeng Qian, Jessica L. Ziehr, Kenneth A. Johnson

**Affiliations:** Institute for Cellular and Molecular Biology, University of Texas at Austin, Austin, TX, USA

**Keywords:** human mitochondria, alpers, Pol-γ mutations, kinetics, yeast, replisome

## Abstract

The human mitochondrial DNA polymerase gamma (Pol-γ) is nuclearly encoded and is responsible for the replication and repair of the mitochondrial genome. Mutations S305R and P1073L in the *POLG* gene have been reported to be associated with early childhood Alpers syndrome. One patient harboring both mutations as compound heterozygous died at 2 years of age after disease onset at 9 months. Quantitative kinetic analysis on purified enzyme showed that the S305R mutation reduces the DNA binding affinity by 10-fold, and reduces the specificity constant (*k*_cat_*/K*_m_) for correct nucleotide incorporation by fourfold. It also causes a ∼threefold reduction in the excision rate to remove mismatched nucleotides. Compared to the wild-type Pol-γ, the S305R mutant showed no product formation in a reconstituted rolling circle replisome assay. Interestingly, the P1073L mutant exhibited wild-type activity in single turnover kinetics to quantify changes in *k*_cat_*/K*_m_, *k*_cat_, *k*_exo_, or processivity, and showed a twofold decrease in the net polymerization rate in the reconstituted replisome assay, while in yeast, P1073L caused a 60–70% mtDNA reduction in haploid cells. The heterozygous diploid yeast cells carrying S305R and P1073L mutations *in trans* showed ∼75% reduction of mtDNA content, relative to homozygous diploid cells with two wild-type alleles. Taken together, we show clearly in both the rolling circle and the humanized yeast system that the P1073L mutation caused significant defects in mtDNA replication, and our results suggest a role for P1073 in the functioning of the Pol-γ with the mitochondrial DNA helicase, and provide a rationale for understanding the physiological consequences of the S305R/P1073L compound heterozygote in humans.

## Introduction

Human mitochondrial DNA is replicated and repaired by DNA polymerase gamma (Pol-γ), which is a heterotrimer composed of a large catalytic subunit (Pol-γA) containing the DNA polymerase active site and the 3′-5′exonuclease for proofreading activity, and two copies of the accessory protein (Pol-γB), which facilitates processive DNA synthesis by improving the ground state nucleotide binding and increasing the rate of chemistry (Johnson et al., 2000; Lee et al., 2009). The Pol-γ holoenzyme functions in conjunction with the mitochondrial DNA helicase (AKA Twinkle; Spelbrink et al., 2001) and single-stranded DNA-binding protein (mtSSB), forming the minimal replisome (Korhonen et al., 2004; Qian et al., 2013).

To date more than 200 mutations have been reported in the *POLG* gene (see http://tools.niehs.nih.gov/polg/) and they are correlated with a variety of mitochondrial disorders, including Alpers syndrome, progressive external ophthalmoplegia (PEO), Parkinsonism, and other encephalomyopathies associated with mtDNA mutations, deletions, and depletions (Stumpf and Copeland, 2011). The progressive, late-onset neurodegenerative diseases (PEO, Parkinsonism, etc.) are often caused by mutations in the active site of Pol-γA (Stumpf and Copeland, 2011), which can lead to reductions in *k*_cat_*/K*_m_, *k*_cat_, and fidelity of replication (Batabyal et al., 2010; Estep and Johnson, 2011; Ziehr et al., unpublished). Reduced fidelity leads to the accumulation of mutations (Qian et al., 2014), while stalling of the polymerase can lead to double strand breaks and DNA depletion (Atanassova et al., 2011; Estep and Johnson, 2011). On the other hand, patients with Alpers syndrome have shown early childhood disease onset, and the symptoms are characterized by intractable epilepsy, psychomotor retardation, and hepatic failure, which leads to early death (Wong et al., 2008; Baruffini et al., 2011; Tang et al., 2011; Farnum et al., 2014). Manifestation of Alpers syndrome typically requires the presence of at least two recessive mutations in Pol-γ, usually in compound heterozygous states, and is mostly associated with mtDNA depletions (Wong et al., 2008). However, the effects of individual recessive mutations on protein function and detailed molecular mechanism of pathogenesis remain to be investigated.

Euro et al. (2011) attempted to cluster Alpers mutations into five postulated functional domains based upon structure and homology to model polymerases. This clustering of mutations into various structural subdomains has been used to rationalize the severity of the diseases according to the expected magnitude of the biochemical defect and to establish guidelines for predicting the clinical effects of new point mutations (Euro et al., 2011; Farnum et al., 2014). However, even within the known functional domains, it is still difficult to predict the magnitude of the biochemical effect of any particular mutation and only a handful of point mutations have been characterized in detail (Batabyal et al., 2010; Estep and Johnson, 2011; Sohl et al., 2013; Ziehr et al., unpublished). Even though several mutations in Pol-γA have been shown to cause reductions in *k*_cat_*/K*_m_, *k*_cat_, processivity, fidelity, and the rate of exonuclease proofreading (Batabyal et al., 2010; Lee et al., 2010; Ziehr et al., unpublished), predicting the physiological consequences of these changes is difficult. Moreover, mutations can affect the interactions between Pol-γA and Pol-γB (Lee et al., 2010), or the TWINKLE and there are currently no reliable assays to quantify the effects of point mutations on these interactions. Predicting the clinical phenotype is further compounded by the presence of a wild-type polymerase allele (or one containing a different mutation), the complex interplay of enzymes during mtDNA replication, and the high mtDNA copy number (Qian et al., 2014).

**FIGURE 1:**
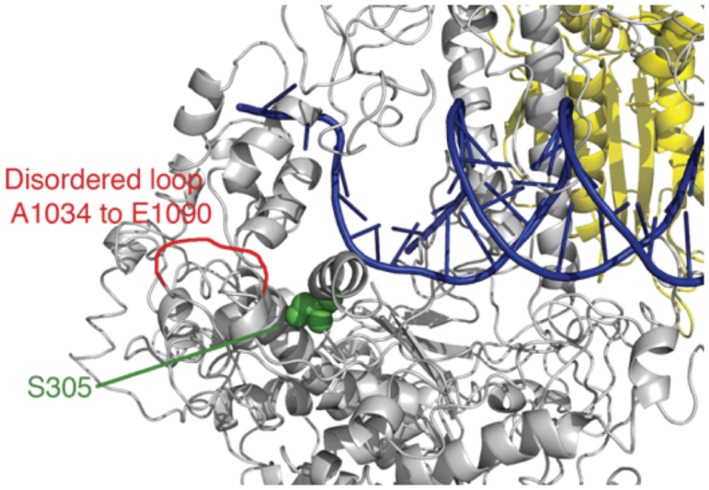
Locations of Ser-305 and Pro-1073. The figure was drawn using 3IKM.pdb with duplex DNA based upon alignment of T7 DNA polymerase (1T7P.pdb).

We previously developed a ‘humanized’ yeast system (Qian et al., 2014) with the yeast Pol-γ MIP1 gene replaced by human Pol-γ, and showed the feasibility of using the ‘humanized’ yeast to study the molecular mechanism of pathogenicity of some human POLG mutations. In the current work, we chose to examine two Alpers mutations: S305R and P1073L (**Figure 1**), respectively, (Wong et al., 2008; Baruffini et al., 2011; Tang et al., 2011; Farnum et al., 2014). One case was reported for a patient carrying both S305R and P1073L mutations as compound heterozygotes (Baruffini et al., 2011). The patient showed onset of Alpers syndrome at 9 months after birth and died at 2 years of age (Baruffini et al., 2011). This rare, severe phenotype may suggest that these two mutant enzymes do not complement each other in mtDNA replication and led to the proposal that Ser-305 and Pro-1073 may be involved in a similar function (Euro et al., 2011). We previously reported that S305R mutant displayed a mild reduction in *k_cat_/K_m_* as measured using single turnover kinetics (Qian et al., 2014). In this work, we quantified the effects of each single point mutation on polymerase activity by Pol-γ alone and with the TWINKLE during DNA polymerization catalyzed by a reconstituted replisome. In addition, we examined the consequences of these two mutations on mtDNA replication in ‘humanized’ yeast cells expressing human Pol-γ (Qian et al., 2014). We showed that S305R and P1073L mutations cause mtDNA replication defects by different kinetic mechanisms, and our findings provided a rationale to understand the physiological consequences of the S305R/P1073L compound heterozygote.

## Materials and Methods

### Purification of Pol-γA and Pol-γB

Wild-type and mutant Pol-γA were expressed in insect cells and purified to homogeneity as described previously (Batabyal et al., 2010). Pol-γB was expressed in *Escherichia coli* and purified to homogeneity as described (Johnson et al., 2000; Qian et al., 2013).

### Cloning, Expression, and Purification of TWINKLE

The human mitochondrial DNA helicase (TWINKLE) with an N-terminal His tag and lacking the first 43 amino acids, was cloned into pcI^ts^ ind^+^ plasmid, and expressed in C2984H cells. The large-scale expression (6 L) was induced at an OD of 4.7 with temperature change to 37°C and addition of Nalidixic acid to a final concentration of 50 mg/ml. The cultures were then grown overnight to a final OD of 13.7 and pelleted at 6,500 ×*g* for 20 min at 4°C. For protein purification, the cell pellet was re-suspended in five volumes (mass to volume) of Lysis buffer (50 mM Tris-HCl pH 7.5, 50 mM NaCl, 20 mM imidazole, 5 mM MgCl_2_.6H_2_O, 0.1 mM PMSF, 10% Glycerol, 0.5% Nonidet-P40) for 20 min. A Dounce A homogenizer was used to ensure uniform suspension. Lysozyme was then added to the suspension to a final concentration of 50 μg/ml and stirred on ice for 15 min. The NaCl concentration was then brought to 500 mM and 1 mM ATP was added to the cell lysate before sonicating for 15 min. This was followed by centrifugation to pellet the debris at 55,000 ×*g* RCF for 30 min at 4°C in a Beckman 45Ti. The supernatant then incubated for 20 min on ice with Ni-Sepharose beads (GE Healthcare) pre-equilibrated with Lysis buffer +1 mM ATP + 500 mM NaCl. The Ni-Sepharose beads were then pelleted at 700 ×*g* RCF for 20 min at 4°C. The pelleted beads and minimal residual supernatant volume were transferred to a jacketed column with water bath set to 4°C and allowed to pack. The column was then washed with 10 column volumes (CVs) of Lysis buffer +1 mM ATP + 500 mM NaCl. Next, the column was washed with five CV of Nickel Column Wash 1 (30 mM Tris-HCl pH 7.5, 20% Glycerol, 0.5% Nonidet-P40, 1 mM β-ME, 5 mM MgCl_2_.6H_2_O, 0.1 mM PMSF, 0.2 mM ATP, 0.5 M NaCl, 20 mM imidazole) followed by a wash with Nickel Column Wash 2 (30 mM Tris-HCl pH 7.5, 20% Glycerol, 0.5% Nonidet-P40, 1 mM β-ME, 5 mM MgCl_2_.6H_2_O, 0.1 mM PMSF, 0.2 mM ATP, 350 mM NaCl, 20 mM imidazole). The protein was then eluted with a linear gradient from 0 to 100% Nickel Column Elution Buffer (30 mM Tris-HCl pH 7.5, 20% Glycerol, 0.5% Nonidet-P40, 1 mM β-ME, 5 mM MgCl_2_.6H_2_O, 0.1 mM PMSF, 0.2 mM ATP, 350 mM NaCl, 250 mM imidazole) over five CV. Fractions were collected and the peak fractions were pooled for further purification on three tandem columns: Q-Sepharose followed by two Heparin Sepharose columns. Columns were washed with 10 CV of Heparin Column Buffer A (30 mM Tris-HCl pH 7.5, 20% Glycerol, 0.5% Nonidet-P40, 1 mM β-ME, 5 mM MgCl_2_.6H_2_O, 0.1 mM PMSF, 350 mM NaCl, 0.2 mM ATP) and the Q-column was removed. The protein was then eluted off of the Heparin Sepharose columns with a steep gradient to Heparin Column Buffer B (30 mM Tris-HCl pH 7.5, 20% Glycerol, 0.5% Nonidet-P40, 1 mM β-ME, 5 mM MgCl_2_.6H_2_O, 0.1 mM PMSF, 1 M NaCl, 0.2 mM ATP). The TWINKLE peak eluted at about 550–600 mM NaCl and fractions were pooled, concentration estimated by Bradford assay, aliquoted, and flash frozen in liquid nitrogen. concentrations of TWINKLE were given in the unit of hexamers, unless otherwise mentioned.

### Cloning, Expression, and Purification of mtSSB

The human cDNA for mtSSB was obtained from OriGene (Rockville, MD, USA). The coding sequence for the gene was examined using MitoProt which indicated that the first 20 amino acids had the highest probability for the mitochondrial targeting sequence (Claros and Vincens, 1996). The mtSSB mature coding sequence (codons 21 through 148) of the gene was amplified by PCR and subcloned into expression vector pcIts ind+ (Brandis and Johnson, 2009), which was transformed into *E. coli* C2984 competent cells to over-express the protein following chemical and temperature induction as described (Brandis and Johnson, 2009). The cell pellets (20 g) were thawed on ice in five volumes of Lysis Buffer (HEPES, pH 7.6; 0.25 mM EDTA; and 1 mM DTT) plus 1 tablet of protease inhibitor cocktails (Roche, NJ, USA). The re-suspended cells were lysed by sonication and cell debris were removed by centrifugation at 1,5000 ×*g* for 30 min at 4°C. The supernatant was loaded onto an Affi-gel Blue column (1.6 cm × 10 cm) equilibrated with Lysis Buffer + 50 mM NaCl at a flow rate of 1 ml/min at 4°C. The affinity column was washed with five CV of Lysis Buffer + 50 mM KCl followed by five CV of Lysis Buffer plus 800 mM NaCl. The mtSSB was eluted with five CV Lysis Buffer plus 0.5 M KSCN. The eluent was collected and protein was precipitated by adding ammonium sulfate to 35% (w/v) saturation and stirring on ice for 1 h. The protein precipitate was collected by centrifugation at 17,000 ×*g* for 15 min at 4°C. The protein pellet was dissolved in 10 ml Dialysis Buffer (25 mM HEPES, pH 7.6; 50 mM NaCl, 0.1 mM EDTA, 2 mM DTT, 10% glycerol) and dialyzed against the same buffer for 4 h at 4°C. The dialyzate was loaded onto a Hi-TRAP SP column (5 ml) equilibrated with Dialysis Buffer, and mtSSB was eluted with a linear 0.05-0.5 M salt gradient. The fractions containing mtSSB to pool were determined by 12% SDS-PAGE to ensure purity (estimated at > 95%). The final concentration of mtSSB was determined using the extinction coefficient ε_280_ = 19,060 M^-1^cm^-1^ for monomers. The concentrations of mtSSB were given in the unit of tetramers, unless otherwise mentioned.

### Nucleotide Incorporation Assays

Single nucleotide incorporation assays were performed with a RQF-3 rapid quench flow instrument (KinTek Corp.) as described (Batabyal et al., 2010; Estep and Johnson, 2011). For a typical nucleotide incorporation assay, 100 nM Pol-γ exo^+^ enzyme was preincubated with 75 nM 5′-γ-^32^P-labeled DNA template (25-mer/45-mer duplex) on ice for 10 min, and the complex was rapidly mixed with 12.5 mM Mg^2+^ and varying concentrations of dATP for variable time at 37°C before the reaction was quenched by mixing with 250 mM EDTA. The products were resolved on a denaturing polyacrylamide sequencing gel, and then the dried gel was exposed to a phosphor screen (Molecular Dynamics). The imaging and quantification were performed as described (Qian et al., 2014). The data have been fit globally using the KinTek Explorer program (KinTek Corp.) to the model shown in Scheme 1, and kinetic parameters *k*_cat_ and *k*_cat_*/K*_m_ were derived for Scheme 1 by the simple relationship *k*_cat_ = *k*_pol_ and *K_m_* = *K*_d,app_.

### Excision Reactions

Enzyme Pol-γ exo^+^ (100 nM) was preincubated with 75 nM DNA containing one T:T mismatch at the 3′ end of the primer. The reaction was initiated by mixing it with Mg^2+^ (10 mM) for variable time at 37°C and was then quenched by 250 mM EDTA. The loss of full-length substrate primer due to exonuclease digestion was plotted against time and fit to a single exponential equation to derive the rate of excision (*k*_exo_).

### Replisome Assays on the Rolling-Circle Template

The 60-mer/70-mer mini-circle DNA substrate was made as described (Qian et al., 2013). The Pol-γ (50 nM) was pre-incubated with 2.5 nM DNA substrate (60-mer/70-mer), 50 nM TWINKLE (hexamer concentration) at 37°C for 30 min in a reaction buffer containing 20 mM Tris-Cl, pH 7.5, 20 mM NaCl, 10 mM MgCl_2_, 3.6 mM ATP, 10% glycerol, 4 mM dithiothreitol, 250 μM dGTP, 50 μM dCTP, 1 μM α-^32^P-dCTP. The reaction mixture was then mixed with 250 μM TTP and 250 μM dATP for 1 min before addition of 500 nM mtSSB tetramer and aliquots were removed at indicated time points and were quenched by 250 mM EDTA. The product was resolved on a 0.8% alkaline agarose gel (12 × 14 cm) containing 50 mM NaOH and 1 mM EDTA at 1.5 v/cm for 20 h. Gels were neutralized by soaking in 6% trichloroacetic acid for 30 min and were dried onto DE81 filter paper (Whatman) and the radioactive bands were detected by PhoshorImaging techniques as described above.

### Processive Polymerization Assays

Processive polymerization by wild-type and the P1073L mutant of Pol-γA was monitored in the rapid quench flow instrument. For the wild-type enzyme assay, 150 nM Pol-γ exo+ enzyme was preincubated with 75 nM 5′-γ-^32^P-labeled DNA template (25-mer primer/73-mer template) for 10 min on ice, before rapidly mixing with 12.5 mM MgCl_2_, 5 μM dATP, 5 μM dGTP, and 5 μM TTP at 37°C for various times and quenching with 0.5 M EDTA. For the P1073L mutant, 150 nM Pol-γ exo+ P1073L enzyme was preincubated with 75 nM 5′-γ-^32^P-labeled DNA template (25-mer primer/45-mer template) for 10 min on ice, before rapidly mixing with 12.5 mM MgCl_2_, 5 μM dATP, 5 μM dCTP, and 5 μM TTP at 37°C for various times and quenching with 0.5 M EDTA. The products of processive polymerization (26-mer to 35-mer for wild-type and 26-mer to 30-mer for P1073L mutant) were resolved by denaturing polyacrylamide gel electrophoresis (15% acrylamide, 8 M urea). The dried gel was exposed to a storage phosphor screen and subsequently scanned and quantified using a Typhoon scanner and ImageQuant software (GE).

### DNA Sequences

25-mer primer: 5′-GCCTCGCAGCCGTCCAACCAACTCA-3′

45-mer template: 5′-GGACGGCATTGGATCGAGGTTGAGT TGGTTGGACGGCTGCGAGGC-3′

73-mer template: 5′-CCCCACCTGCAGGCATGCAAGCTTGG CACTGGCCGTCGTTTTACCTCTTGAGTTGGTTGGACGGC TGCGAGGC-3′

### Humanized Yeast Strains

The haploid cells with the MIP1 replaced by Pol-γ ^S305R^ or Pol-γ ^P1073L^ were constructed using the genomic targeting technique as described previously (Qian et al., 2014). The heterozygous diploid cells were constructed by mating between the MATα type of haploid strains (*mip1*::Pol-γ^P1073L^) with the MATa type of haploid strains (*mip1*::Pol-γ^S305R^) as described previously (Qian et al., 2014).

### Miscellaneous Methods

Methods for measurement of petite frequency, mutation frequency, mtDNA contents, and growth curve analysis were described previously (Qian et al., 2014).

## Results

### Pre-steady-State-Burst Kinetics

**FIGURE 2:**
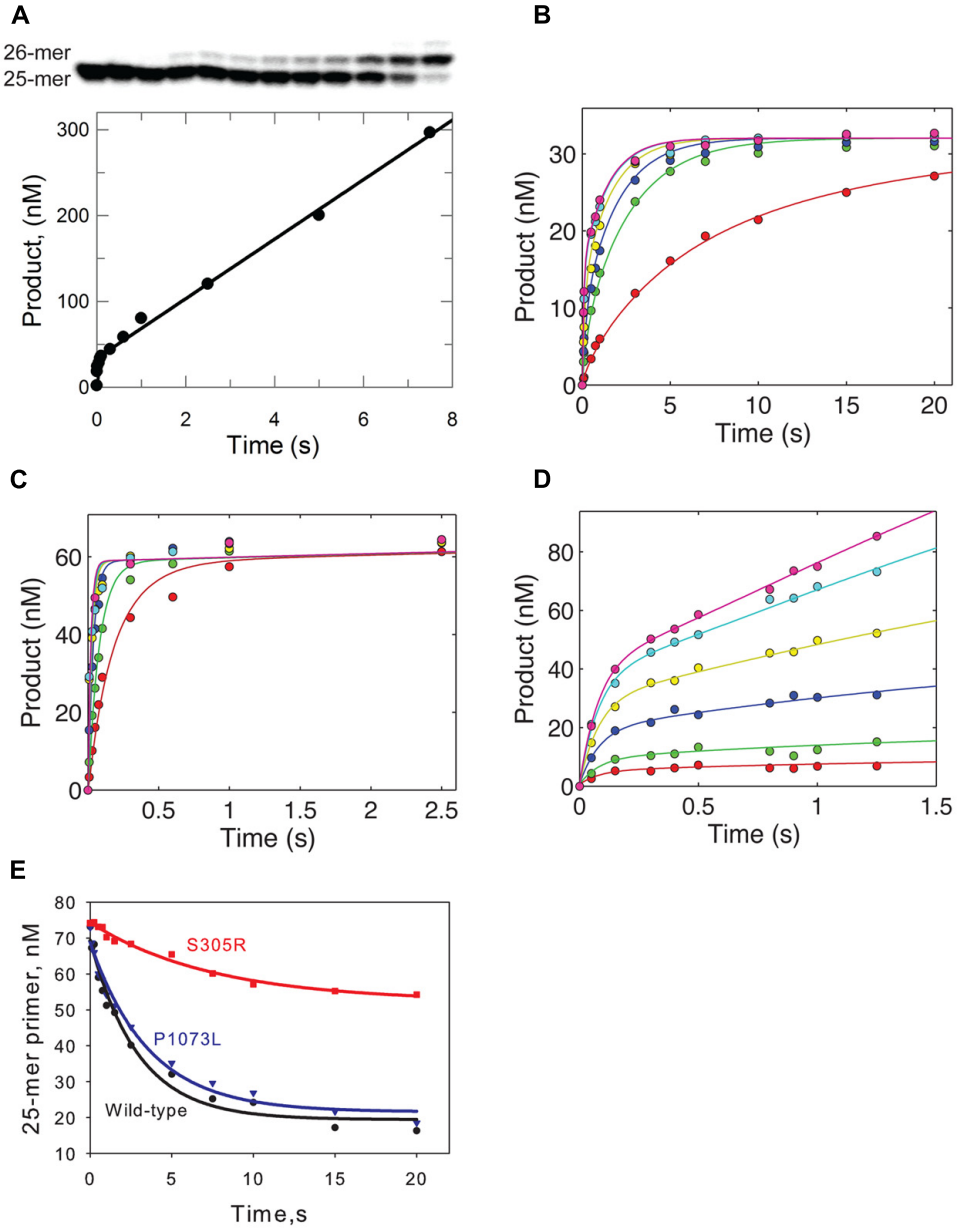
Kinetic analysis of S305R and P1073L mutant. **(A)** Active site titration of S305R mutant. The S305R mutant (100 nM) was preincubated with 350 nM DNA template (25-mer/45-mer) for 10 min on ice. The binary complex was then rapidly mixed with 10 mM Mg^2+^ and 50 μM dATP (correct incoming nucleotide). The reaction was allowed to proceed for variable times before quenched by 250 mM EDTA. The product was resolved on a 15% denaturing polyacrylamide gel as shown in the upper panel, quantified and plotted against the reaction time in the lower panel. The data were fit to a burst equation as indicated in the text. **(B)** Kinetic incorporation of dATP for S305R mutant and P1073L mutant **(C)**. For each concentration series, a preformed enzyme-DNA complex ([enzyme] > [DNA]) was mixed with variable concentrations of nucleotide (0.05, 0.2, 0.4, 1, 6, and 17 μM) for S305R mutant **(B)** and (0.2, 0.5, 1.5, 3, 5, and 10 μM) for P1073L mutant **(C)**, respectively, and then quenched with 250 mM EDTA. Some of data shown in **(B)** were collected from (Qian et al., 2014). In each panel, the smooth lines represent the best fit to the model shown in Scheme 1 derived using KinTek Explorer software. The rate constants were determined and shown in . The error analysis of data collected for the S305R mutant was shown in **Table 1**. **(D)** DNA concentration-dependent kinetic incorporation of dATP for S305 mutant. The enzyme (55 nM) was preincubated with variable concentration of DNA substrate (10, 30, 50, 100, 200, and 300 nM) and was mixed with 100 μM dATP and 12.5 mM Mg^2+^ for varying time as indicated in the figure. **(E)** Excision of DNA containing a single T–T mismatch at the 3′-terminal of the primer. Enzyme (100 nM) was preincubated with 75 nM DNA substrate (25-mer/45-mer), and Mg^2+^ and excess unlabeled DNA were added to initiate the cleavage reaction. The remaining 25-mer was plotted against time and fit to a single exponential to yield an excision rate of 0.34 ± 0.04 s^-1^ for wild-type enzyme, 0.11 ± 0.02 s^-1^ for S305R mutant, and 0.28 ± 0.03 s^-1^ for P1073L mutant.

For wild-type Pol-γ, dissociation of the DNA from the enzyme-DNA binary complex is the rate-limiting step during steady state single turnover experiments, and the rate of this step (*k*_off_) governs the steady-state rate (*k*_ss_). To determine whether the mutation affects the steady-state rate, primer extension assays were performed with a total enzyme concentration of 100 nM and 350 nM DNA template (25-mer/45-mer). After pre-incubation to form a binary complex, reactions were started by the addition of correct incoming nucleotide (dATP) to a final concentration of 50 μM and product (26-mer) formation was allowed up to 8 s. The amount of product was quantified, plotted against time, and fit to a burst equation ([product] = A ⋅ (1-e^-kt^)k_ss_ ⋅ t) to obtain the steady-state rate (*k*_ss_) during the single nucleotide incorporation (**Figure 2A**). The *k*_off_ was derived by the equation (*k*_off_ = *k*_ss_/A), where A is the concentration of active enzyme-DNA complex. Compared to the wild-type enzyme, the S305R mutant shows a 60-fold increase of *k*_off_ from 0.02 s^-1^ (Johnson et al., 2000) to 1.2 s^-1^. This suggests the Ser-305 residue is involved in DNA binding, which is consistent with its location near the DNA binding pocket (**Figure 1**). The P1073L mutant showed a *k*_off_* -*value similar to the wild-type enzyme (data not shown).

### Single-Nucleotide Incorporation Kinetics

The enzyme was pre-incubated with DNA template (25-mer/45-mer) to form the binary complex, and it was rapidly mixed with a solution containing the correct incoming nucleotide (dATP) at various concentrations. The nucleotide concentration dependence of the rate of the rapid burst of polymerization provided estimates of an apparent nucleotide dissociation constant (*K*_d,app_) and a maximum rate of nucleotide incorporation (*k*_pol_) according to Scheme 1 as described previously (Batabyal et al., 2010). **Figures 2B,C** shows the time course of product formation (26-mer) at several nucleotide concentrations observed for S305R and P1073L mutants, respectively. In **Figure 2B**, some data were taken from **Figure 4A** published previously (Qian et al., 2014) with the addition of data collected from experiments using lower concentrations of dATP. The data defining nucleotide incorporation were fit globally to the mechanism shown in Scheme 1 to obtain the *k*_pol_ and *K*_d,app_.

$$E+D_{25}\overset{k_{1}}{\underset{k_{-1}}{⇄}}{E.D}_{25}$$

$${E.D}_{25}+N\overset{k_{2}}{\underset{k_{-2}}{⇄}}{E.D}_{25}.N\overset{k_{3}}{\to }E.D_{26}+PP_{i}$$

$$E+D_{26}\overset{k_{1}}{\underset{k_{-1}}{⇄}}{E.D}_{26}\,\;\;\;\;\;\;\;\;\;\;\left(Scheme\;\;1\right)$$

Where D_25_ represents the DNA substrate (25-mer/45-mer) and E.D_25_ represents a preformed complex of enzyme (E) with DNA. Ground state nucleotide (N) binding is represented by the term *K*_d,app_, which can be derived by *K*_d,app_ = *k_-2_*/*k_2_*. The nucleotide association rate (*k*_2_) is diffusion limited and was fixed at 0.5 nM^-1^s^-1^ for global data fitting, whereas chemistry step to form the complex of enzyme with 26-mer/45-mer (E.D_26_) and pyrophosphate (PPi) is irreversible and described by *k*_pol_ and *k*_pol_ = *k*_3_. The DNA dissociation rate is described by *k*_off_ and *k*_off_ = *k*_-1_, whereas the DNA association rate is *k*_on_ and *k*_on_ = *k*_1_.

According to the simplified model, the polymerization rate is governed by a single rate-limiting step (*k*_pol_), and the ground state nucleotide binding occurs as a fast equilibrium, while pyrophosphate release and translocation appear to be fast (Batabyal et al., 2010). Therefore, the measured value of *k*_pol_*/K*_d,app_ defines *k*_cat_*/K*_m_, the specificity constant governing nucleotide incorporation during processive polymerization. Interestingly, the P1073L mutant showed a maximum rate of polymerization (**Figure 2C**) and the value of *k*_pol_*/K*_d,app_ comparable to the wild-type enzyme as summarized in **Table 1**. For the S305R mutant, a plot of concentration of product with respect to the reaction time was biphasic in nature (**Figure 2B**), displaying a fast nucleotide incorporation reaction followed by slower subsequent reactions. The faster phase represents the incorporation of dATP in a single turnover reaction. As the reaction proceeds, the slower phase is rate-limited by the re-equilibration of enzyme-DNA binary complex.

**Table 1:** Effects of S305R and P1073L mutations on dATP incorporation.

	*K_*d,DNA*_* nM	*k_*pol*_* s^-1^	*K_*d,app*_* μM	*k_*pol*_/K_*d,app*_*	μM^-1^s^-1^	*k_*exo*_*s^-1^
WT^∗^	9.9 ± 2.1	30 ± 2	0.7 ± 0.14	43 ± 9	0.34 ± 0.04
S305R	100 ± 4	12.4 ± 0.75	1.2 ± 0.15	10 ± 1	0.11 ± 0.02
P1073L	10.2 ± 1.2	54.2 ± 11.8	1.5 ± 0.5	36.1 ± 7	0.28 ± 0.03

^*^The kinetic parameters for WT enzyme were determined previously (Johnson et al., 2000; Batabyal et al., 2010).

To accurately determine the effect of S305R on DNA binding affinity, we performed another single nucleotide incorporation assay, where the enzyme concentration was fixed at 55 nM, and the final concentration of DNA was varied between 10 and 300 nM. The time dependence of product (26-mer) formation for a representative set of DNA concentrations is shown in **Figure 2D**. The data were then analyzed by fitting the full time course to the model (Scheme 1). Global fitting of data shown in **Figures 2B,D** defines the DNA association rate (*k*_on_), *k*_off_, *k*_pol_, and *k*_cat_*/K*_m_ for S305R mutant. As shown in **Table 1**, the S305R mutation caused a 2.5-fold reduction in *k*_pol_, a fourfold reduction in *k*_cat_*/K*_m_, and an overall 10-fold decrease in DNA-binding affinity.

### Exonuclease Activity of S305R and P1073L Mutant

The Ser305 is located near the exonuclease active site of Pol-γA (**Figure 1**). To determine whether the mutation affects the proofreading activity of Pol-γ, we examined the excision of the 3′-terminal base from the primer strand of DNA containing one T:T mismatch. Cleavage was examined by mixing Mg^2+^ and excess unlabeled DNA as a polymerase trap with a pre-formed enzyme-labeled DNA binary complex. The fraction of remaining primer (25-mer) was plotted against the reaction time (**Figure 2E**), and the data were fit to a single exponential equation. The S305R mutation reduces the rate of excision of T:T mismatch from 0.34 to 0.11 s^-1^, while the P1073L mutation appears to have no impact on the exonuclease activity within the experimental uncertainty (**Table 1**).

### Confidence Contour Analysis

**FIGURE 3:**
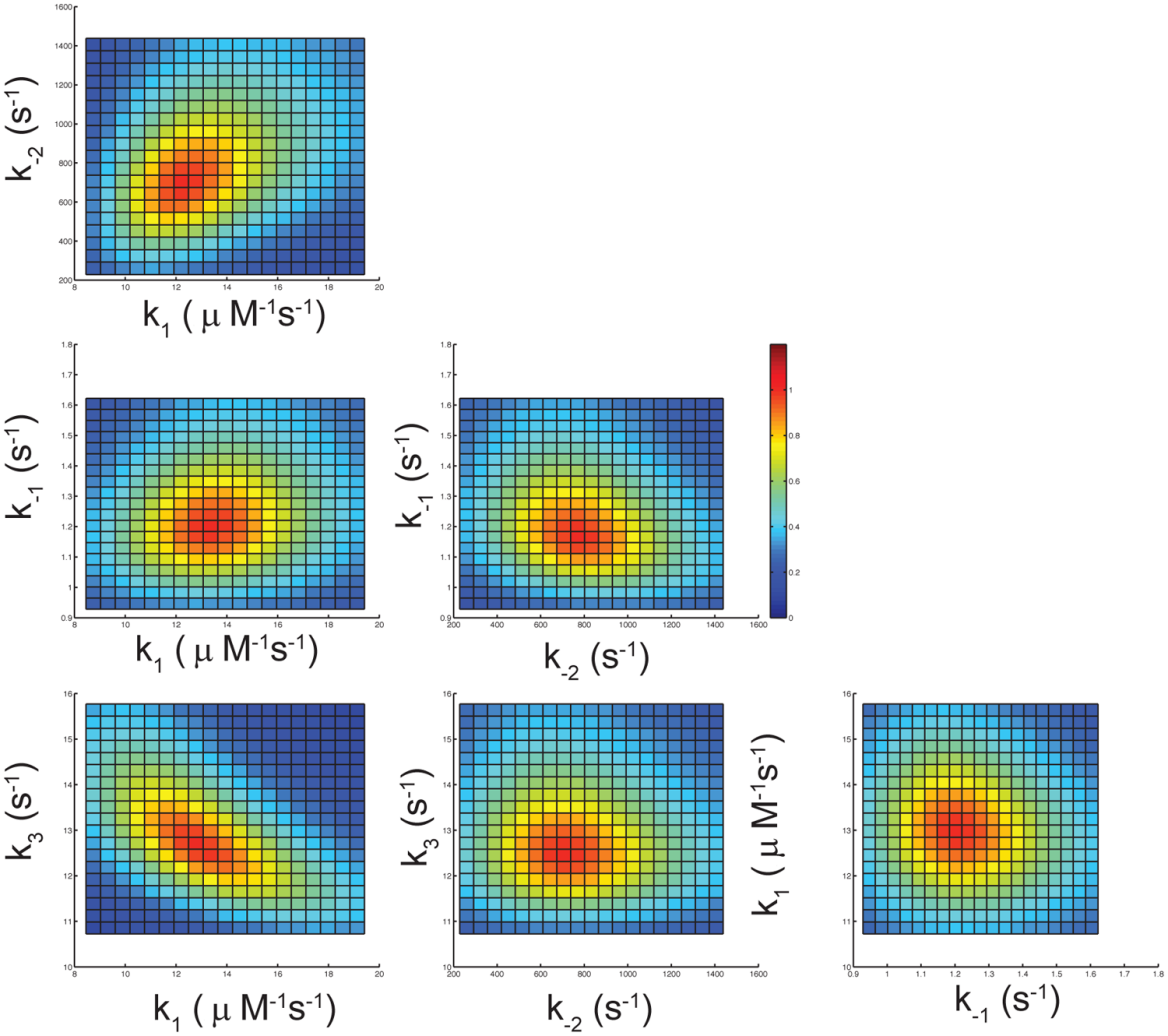
Confidence contour analysis. The error limits of the four rate constants governing the kinetic pathway of nucleotide incorporation by S305R mutant, were investigated by confidence contour analysis of pair wise combinations of the four rate constants as described previously (Johnson et al., 2009a,b). The results were derived by globally fitting the data as shown in **Figures 2B,D** and were displayed as a normalized χ^2^-values as a function of each parameter. The central red zone shows the area of good fit, and the yellow band between the red and green zones shows the threshold representing a 10% increase in χ^2^ (Johnson et al., 2009b).

To determine whether the data collected in **Figures 2B,D** are sufficient to define four kinetic parameters (*k*_1_, *k*_-1_, *k*_-2_, and *k*_3_) in fitting to Scheme 1, a confidence contour analysis was performed as described previously (Johnson et al., 2009a,b). **Figure 3** shows the normalized χ^2^-values as a function of each parameter. The results demonstrate all four kinetic parameters were well constrained by the data as indicated by the red central zone defining the area of good fit. The yellow margin shows the threshold representing a 10% increase in χ^2^, which is used to set the lower and upper confidence limits on each of the kinetic parameters.

### Processive Polymerization by Wild-Type and P1073L Pol-γ

**FIGURE 4:**
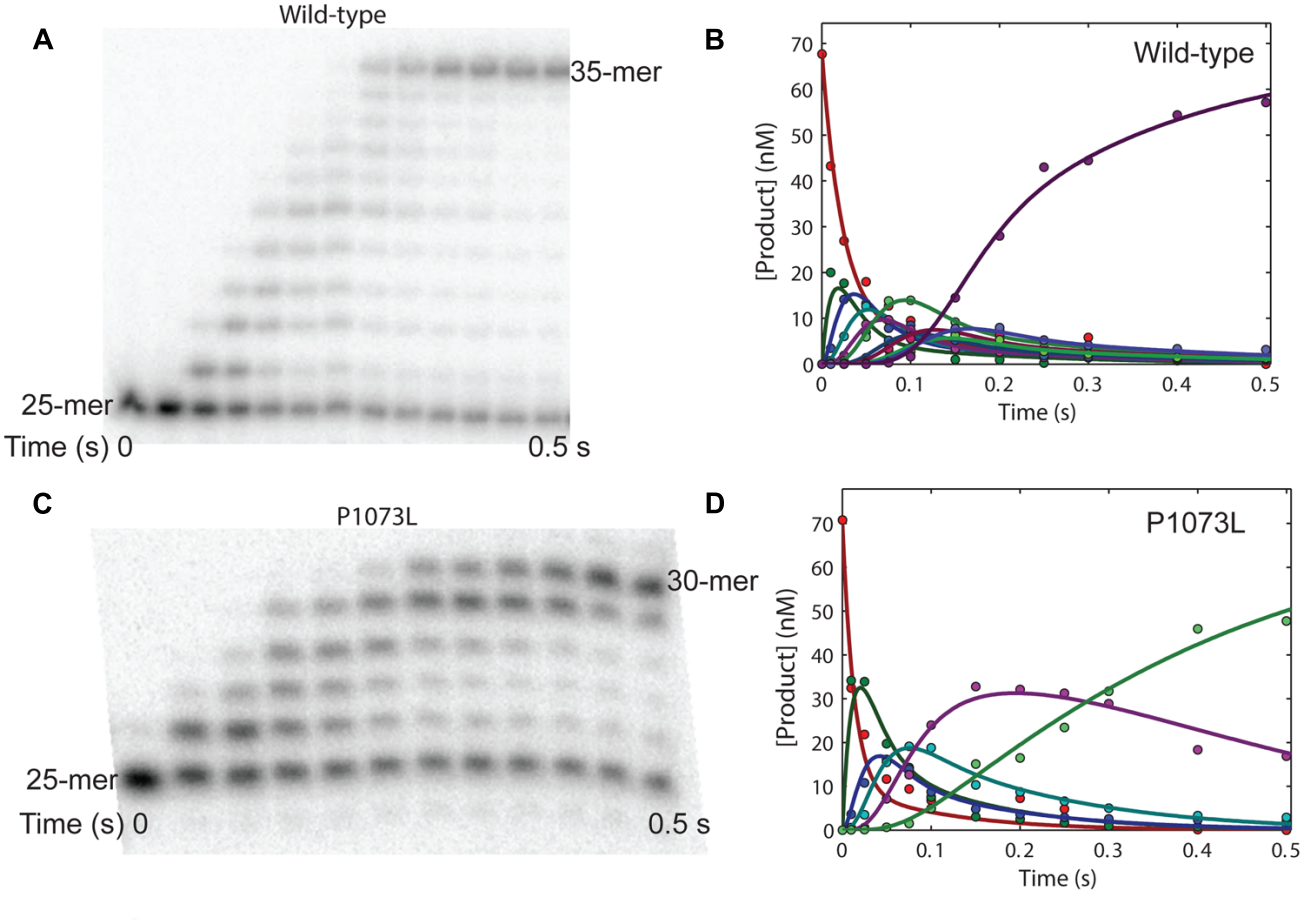
Processive polymerization by wild-type and mutant P1073L polymerase gamma (Pol-γ). **(A)** A preincubated solution of wild-type Pol-γ and 25/73mer DNA was reacted with dATP, dGTP, TTP (5 μM each) in buffer containing 12.5 mM MgCl_2_ for various times before quenching with 0.5 M EDTA and analyzing by polyacrylamide gel electrophoresis (15%, 8 M urea). **(B)** The curves were obtained by quantifying the amount of 25-mer through 35-mer over time as the fractional concentration of each species at each time point multiplied by the concentration of DNA. The solid lines represent the global fit of the data to a model for processive incorporation of 10 single nucleotide incorporations at rates of 37, 75, 60, 65, 70, 37, 73, 58, 70, and 46 s^-1^ and a *K*_d,DNA_ = 15 nM. **(C)** A preincubated solution of P1073L Pol-γ and 25/45mer DNA was reacted with dATP, dCTP, TTP (5 μM each) in buffer containing 12.5 mM MgCl_2_ for various times before quenching with 0.5 M EDTA and analyzing by polyacrylamide gel electrophoresis (15%, 8 M urea). **(D)** The curves were obtained by quantifying the amount of 25-mer through 30-mer over time as the fractional concentration of each species at each time point multiplied by the concentration of DNA. The solid lines represent the global fit of the data to a model for processive incorporation of five single nucleotide incorporations at rates of 65, 36, 48, 28, and 7 s^-1^ and a *K*_d,DNA_ = 15 nM.

The P1073L mutant shows wild-type activity in single-turnover kinetics. We next examined the processivity of P1073L mutant on a 25/45-mer DNA template. As shown in **Figure 4C**, a preincubated solution of P1073L Pol-γ and 25/45-mer DNA was reacted with dATP, dCTP, TTP (dGTP was absent so that only five nucleotides were allowed to incorporate) for various times before quenching with 0.5 M EDTA and analyzing by polyacrylamide gel electrophoresis. The curves (**Figure 4D**) were obtained by quantifying the amount of 25-mer through 30-mer over time as the fractional concentration of each species at each time point multiplied by the concentration of DNA. The solid lines represent the global fit of the data to a model for processive incorporation (Scheme 2) of five single nucleotide incorporations at rates of 65 s^-1^ (dATP), 36 s^-1^ (dCTP), 48 s^-1^ (dCTP), 28 s^-1^ (TTP), and 7 s^-1^ (dCTP), and a K_d,DNA_ = 15 nM. The wild-type enzyme (**Figures 4A,B**) shows processive incorporation of 10 single nucleotide incorporations at rates of 37, 75, 60, 65, 70, 37, 73, 58, 70, and 46 s^-1^ and a K_d,DNA_ = 15 nM on a 25/73-mer DNA template. The discrepancy between polymerization rates could be base-dependent and sequence-context dependent. Overall, the P1073L mutant shows no apparent defect in processive DNA synthesis. equation

$$ED_{25}\overset{k_{pol1}}{\to }ED_{26}\overset{k_{pol2}}{\to }ED_{28}\overset{k_{pol3}}{\to }ED_{28}\overset{k_{pol4}}{\to }ED_{29}\overset{k_{pol5}}{\to }ED_{30}$$

$${ED}_{n}\overset{K_{d}}{⇄}E+D_{n}\,\;\;\;\;\;\;\;\;\;\;\;\;\;\;\;\;\;\;\;\;\;\;\;\;\;\;\;\;\;\;\;\;\;\;\;\;\;\;\left(Scheme\;\;2\right)$$

### Replisome Polymerization Kinetics of S305R and P1073L Mutant

**FIGURE 5:**
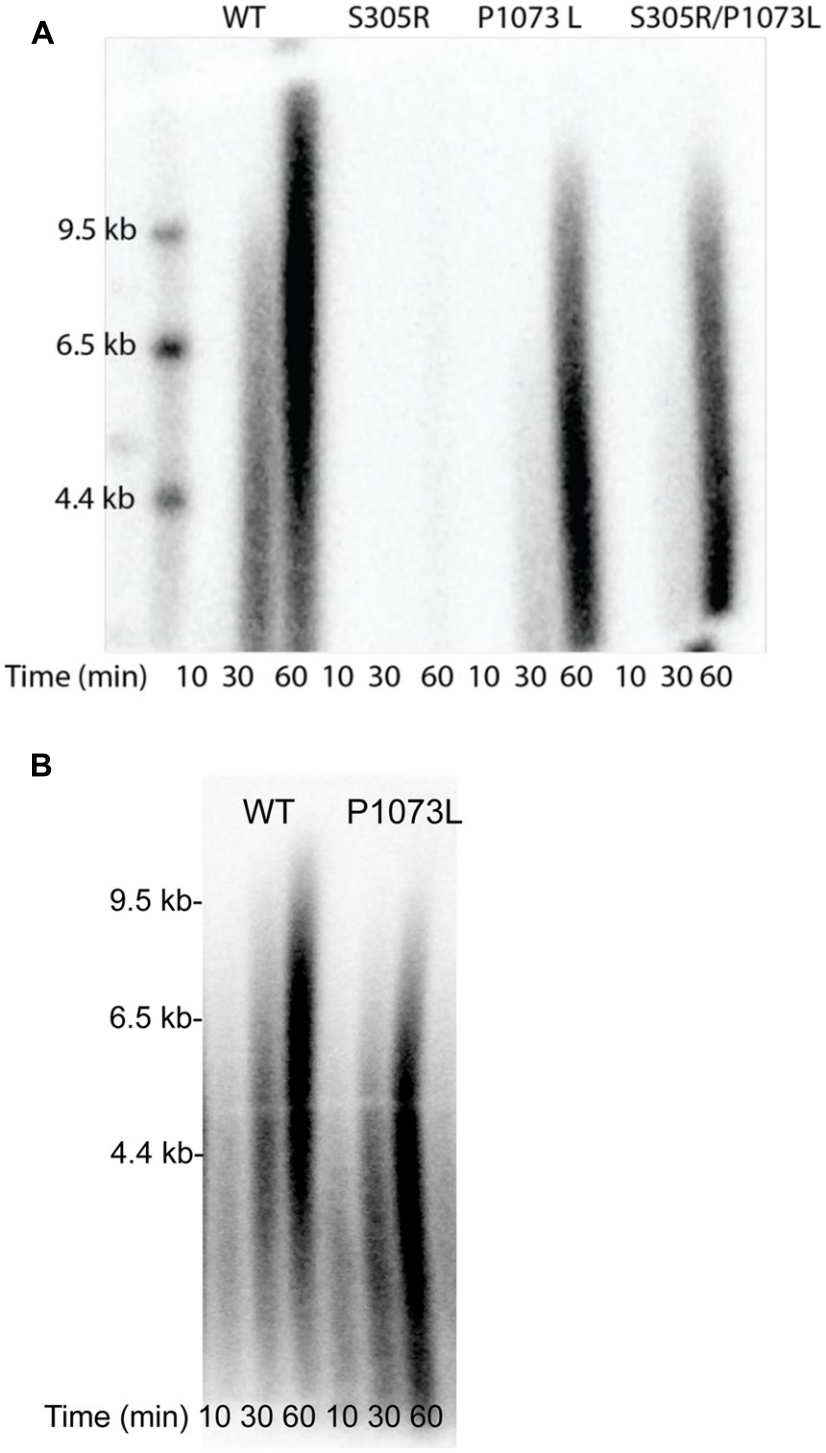
Rolling-circle DNA replication by the reconstituted mtDNA replisome. Results are shown for WT, S305R, P1073L, and the combination S305R/P1073L mutants. Size markers are given in kb. The reaction was run using 50 nM Pol-γ (WT or mutant), 50 nM TWINKLE, and 0.5 μM mtSSB **(A)** or without mtSSB **(B)**, added after 60 s, in the presence of 250 μM of each of the four dNTP’s and 3.6 mM ATP. Products formed after 10, 30, and 60 min were resolved on an alkaline agarose gel to get results shown.

Our attempts characterizing the enzyme activity for P1073L mutation in Pol-γA catalytic subunit have failed to reveal a measureable defect. Pro-1073 is located in a disordered loop in the published structure (**Figure 1**). The presumption is that P1073 may reflect interactions between Pol-γ and other components of the replisome, notably the TWINKLE. To test this, we have assembled a minimal replisome containing Pol-γ (50 nM), TWINKLE (50 nM), and mtSSB (500 nM) on a mini-circle template (2.5 nM) to examine leading-stranding synthesis (Qian et al., 2013). The wild-type enzyme shows long products being synthesized through rolling-circle replication, while no products were seen with the S305R mutant (**Figure 5A**). The data are consistent with our previous observation that the S305R mutant failed to support replication in haploid yeast cells (Qian et al., 2014). Interestingly, the P1073L mutation caused approximately a twofold reduction in the net polymerization rate in the replisome assay. To examine whether the defect is due to the impaired interaction between Pol-γ and mtSSB, we repeated the experiment by excluding the mtSSB in the reaction, similar results were obtained as shown in **Figure 5B**, suggesting that P1073L mutation may disturb the interaction between Pol-γ and TWINKLE.

To approximate the compound heterozygote seen clinically, we mixed equal parts of the two mutant enzymes to get the results in the column labeled S305R/P1073L. Under these conditions, the S305R mutant only slightly reduced the net rate of polymerization catalyzed by the P1073L mutant. This result can be explained by the weaker DNA binding affinity of S305R mutant so that the S305R mutant does not interfere with the polymerization catalyzed by the P1073L mutant.

### Effects of S305R and P1073L Mutation on mtDNA Replication in Humanized Yeast

**FIGURE 6:**
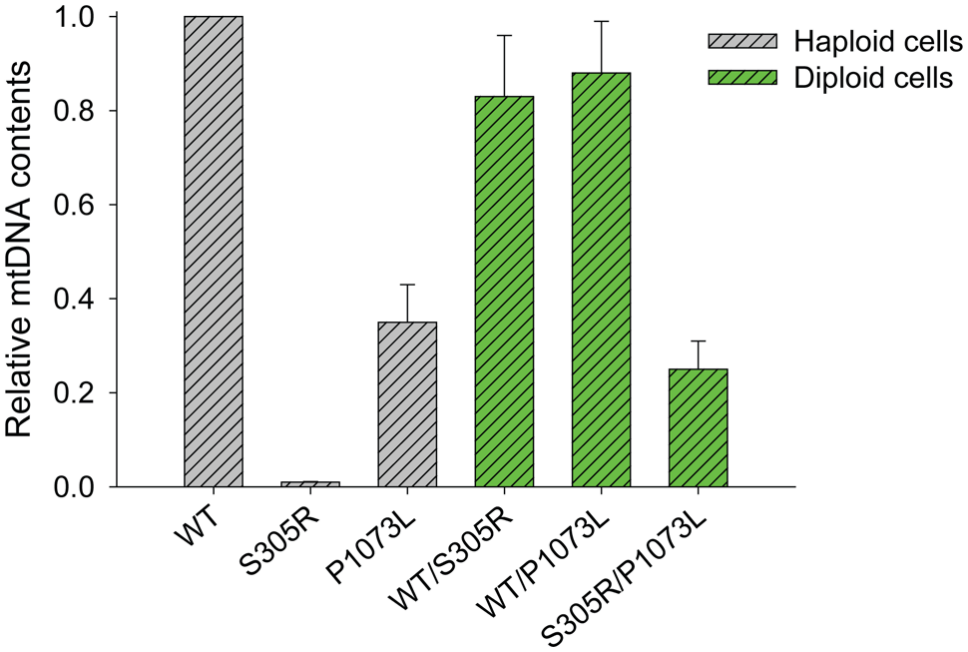
Relative mtDNA content of different humanized yeast strains. The copy number of the mitochondria *COX3* gene relative to that of the nuclear *GAL4* gene by q-PCR was normalized to 1 for POLG(WT) haploid strain and POLG(WT)/POLG(WT) diploid strain as described previously (Qian et al., 2014).The genomic DNA was extracted from cells harvested at OD = 1.0. The data for S305R haploid and S305R/WT diploid cells were determined previously (Qian et al., 2014).

We have shown in our previous work (Qian et al., 2014) that human Pol-γ can complement yeast MIP1 knockouts in mitochondria maintenance. Yeast carrying the human enzyme with an S305R mutation in Pol-γ showed a complete loss of mtDNA content in haploid cells, while in diploid cells (with one wild-type allele), we observed a decreased growth rate, correlating with loss of mtDNA content and increased mtDNA mutation frequency over time (Qian et al., 2014). In comparison, yeast haploid cells expressing Pol-γ (P1073L) retained 30–40% of mtDNA content relative to that of cells expressing the wild-type Pol-γ (**Figure 6**), but the P1073L strain showed ∼fourfold increase in mtDNA mutation frequency determined by the percentage of cells resistant to erythromycin (**Table 2**). In both heterozygous diploid strains: WT/S305R and WT/P1073L, the mtDNA contents retained at least 80% relative to the WT/WT diploid strains (**Figure 6**), suggesting the wild-type enzyme can compensate for deficiencies in the rates of replication catalyzed by the mutant forms. However, both heterozygous diploid cells showed elevated mtDNA mutation frequency: ∼eightfold increase for WT/S305R diploid cells and ∼fourfold increase for WT/P1073L diploid strains. Diploid cells carrying S305R/P1073L mutations to mimic the compound heterozygotes of human Pol-γ mutations, showed more dramatic mitochondrial deficiency with ∼80% reduction of mtDNA content, and ∼25-fold increase of mtDNA mutation frequency (**Figure 6** and **Table 2**).

**Table 2:** mtDNA mutation frequency as measured by Ery*^R^* cells.

Strains	Ery*^R^*(10^-7^)
POLG(WT)	2.3 ± 0.2
POLG(S305R)	Not available
POLG(P1073L)	7.2 ± 1.1
POLG(WT)/POLG(WT)	1.5 ± 0.5
POLG(WT)/POLG(S305R)^∗^	9 ± 1.1
POLG(WT)/POLG(P1073L)	4.4 ± 0.6
POLG(S305R)/POLG(P1073L)	24 ± 2.1

*^∗^Data was collected previously (Qian et al., 2014)*.

## Discussion

There is a large gap in our understanding of the structure/function relationships underlying mtDNA replication. With a given mutation one would like to define the changes in kinetics of polymerization and to understand how biochemical defects lead to the observed phenotype. In particular it is important to understand the extent to which a wild-type enzyme compensates for a defective polymerase, what attributes cause a mutant enzyme to interfere with the functioning of a wild-type enzyme, and how different mutations in compound heterozygotes interact to produce more severe phenotypes. In this work, we started to address these questions by characterizing two Alpers mutations S305R and P1073L, using complementary approaches including examining the effect of mutations on DNA polymerization efficiency (*k*_cat_*/K*_m_), fidelity, and the net DNA replication efficiency in a reconstituted replisome, and by monitoring the physiological effect of mutations in a humanized yeast with the endogenous yeast mitochondrial DNA polymerase (MIP1) replaced by human Pol-γ.

Serine 305 lies in a loop-helix motif adjacent to a disordered loop (residues A1034 to E1090) in the exonuclease domain (**Figure 1**); it is noteworthy that residues in this motif are highly conserved from yeast MIP1 to human Pol-γ (Szczepanowska and Foury, 2010). Szczepanowska and Foury (2010) analyzed the biochemical defects of equivalent mutations (L304R, R309H, R309L, and W312R) in this motif in yeast MIP1 and all variants exhibited reduced DNA-binding affinity. Our data show that the human Pol-γ (S305R) mutant had a 10-fold reduction in DNA-binding affinity, which is primarily caused by a faster DNA dissociation rate (**Figure 2A**). Thus, the primary effect of the S305R mutation on the polymerase activity is attributed to the reduction of the processivity as determined by *k_pol_*/*k_off_* from ∼2000 nt (wild-type enzyme) to ∼10 nt. Consistently, the S305R mutant failed to synthesize detectable long DNA in our reconstituted replisome assay (**Figure 5**), and yeast haploid strain carrying S305R mutation showed 100% *petite* phenotype (Qian et al., 2014) and a completely loss of mtDNA content. In humans diagnosed with Alpers syndrome, the S305R mutation is always found *in trans* with another mutation (A467T, P1073L, or R627Q; Baruffini et al., 2011; Tang et al., 2011; Farnum et al., 2014). The A467T mutation has been shown to reduce the DNA-binding affinity due to the compromised interaction between Pol-γA and Pol-γB (Chan et al., 2005). Thus, it can be predicted that neither S305R nor the A467T mutant is able to efficiently utilize the DNA template for processive mtDNA replication *in vivo*, which leads to mtDNA depletion that is commonly seen in patients with Alpers syndrome (Wong et al., 2008).

The molecular mechanism of the pathogenicity of S305R/P1073L and S305R/R627Q compound heterozygote mutations, nevertheless, is likely to be different from S305R/A467T mutations. Kinetic analysis of the purified P1073L enzyme shows wild-type behavior in the DNA-binding affinity, processivity, *k*_cat_*/K*_m_ for correct nucleotide incorporation and proofreading activity (**Figures 2** and 4). Likewise, the R627Q mutant (Luoma et al., 2005) exhibits normal DNA polymerase activity, and slightly higher DNA-binding affinity and processivity. Our experimental data do not support the previous proposal to cluster Alpers mutations based on structure-function relationship based upon domain location alone (Euro et al., 2011), which suggests that Pro-1073 and Ser-305 belong to the same cluster and are involved in the same function involving the partitioning of the primer strand between the *pol* and *exo* active site by forming stable contacts with the DNA substrate (Euro et al., 2011). Rather, the effect of the P1073L mutation appears to be subtler.

Based upon available polymerase assays alone, one would have been tempted to dismiss claims that the P1073L mutation (or R627Q) was correlated genetically with mitochondrial disorders. Here we show clearly in both the rolling circle assays and the humanized yeast system that the P1073L mutation caused significant defects in mtDNA replication (**Figures 5** and **6**) through mechanisms that remain to be established. Our results suggest a role for Pro-1073 in the functioning of the Pol-γ with the TWINKLE, based on its location in a disordered loop in the Pol-γ structure (**Figure 1**), but more definitive assays are needed to quantify this effect. Mutations in other residues in this disordered loop, such as Alpers mutation R1074W, as well as PEO mutations G1051R and G1076V, cause mtDNA instability to a different extent in the yeast system (Baruffini et al., 2007; Stumpf et al., 2010), arguing the important role of this loop. Nevertheless, detailed biochemical characterization of these mutants is needed in order to fully understand the function of this loop.

Under physiological conditions, the inter-play between two alleles primarily determines the consequences of mutations. Our data shown in this work, as well as studies from other work (Stumpf et al., 2010; Baruffini et al., 2011; Qian et al., 2014), demonstrate that the wild-type allele can largely compensate for the defects caused by the slow polymerization rate of a mutant allele (**Figure 6**), and that the onset of growth defects is likely due to the increased mutation frequency (**Table 1**). In fact, aging-dependent accumulation of point mutations in various regions of mtDNA were reported in human studies (Stumpf et al., 1999; Wang et al., 2001; Bender et al., 2006), and the important role of mtDNA mutation to disease progression is also supported by the mouse model carrying the proofreading-deficient Pol-γ in several studies (Trifunovic et al., 2004; Kujoth et al., 2005; Ross et al., 2013), which led to premature aging phenotype. The significance of mtDNA mutation in disease progression of Alpers syndrome, was considered to be limited (Stumpf et al., 2010), as compared to the impact of slow replication rates catalyzed by mutant enzymes, and resulting in mtDNA depletion as shown in several tissues of Alpers patients (Naviaux et al., 1999; Naviaux and Nguyen, 2004). Consistently, the primary effect of S305R/P1073L in compound heterozygous states is reflected by ∼80% reduction in mtDNA content. Nevertheless, it is interesting to note that the S305R/P1073L diploid strain showed ∼fivefold increase of mtDNA mutation frequency (**Table 2**) compared to the WT/P1073L diploid strain, arguing for a role of mtDNA mutation in mitochondrial dysfunction and disease progression, which may be caused by the low fidelity S305R enzyme (**Table 2**).

Overall, we present evidence showing different molecular and kinetic mechanisms causing Alpers disease by S305R and P1073L mutants. To our knowledge, this is the first report that suggests an important role of Pro-1073 in coordinating with other replisome components in processive mtDNA replication, although further experiments are needed to test this postulate. We conclude that characterizing the deficiency of Pol-γ mutants in the context of replisome as a whole is important for understanding mitochondrial disease and for predicting disease severity.
